# Life stressors and resources as predictors of adolescent suicide attempt

**DOI:** 10.1192/j.eurpsy.2021.460

**Published:** 2021-08-13

**Authors:** E. Du Plessis

**Affiliations:** Psychology, University of the Free State, Bloemfontein, South Africa

**Keywords:** life stressors, adolescence, Suicide, ethnic

## Abstract

**Introduction:**

Adolescent suicide poses a serious public health challenge. Several factors, such as early losses, discordant relationships, poverty, abuse and other life crises have previously been associated with the rise in adolescent suicides. However, a dearth of information exists regarding South African research on adolescent suicide.

**Objectives:**

This study investigated the role of gender, race and psychosocial stressors and resources in attempted suicide among 1033 South African adolescents.

**Methods:**

Using a cross-sectional research design, participants completed a biographical questionnaire and the Life Stressors and Social Resources Inventory, Youth Form. Logistic regression analysis was used to identify which stressors, resources and demographic variables, best predicted attempted suicide among the sample.

**Results:**

The findings suggest that 12.5% (129) of the sample had previously attempted suicide. Being of mixed race (p ≤ .01) and female (p ≤ .01) significantly increased the likelihood of attempting suicide. Stressors associated with the increased likelihood of attempting suicide were Parents (p ≤ .05), Extended Family (p ≤ .01), Home and Money (p ≤ .05), and Negative Life Events (p ≤ .01). Resources associated with the reduced likelihood of attempting suicide were supportive relationships with Parents (p ≤ .01), with Boyfriend/Girlfriend (p ≤ .01) and Positive Life Events (p ≤ .01).

**Conclusions:**

These findings highlight the importance of supportive relationships and stable home conditions for the well being of adolescents.

**Disclosure:**

No significant relationships.

